# Inorganic Chemistry

**Published:** 1886-04

**Authors:** 


INORGANIC CHEMISTRY. *By* EDWARD FRANKLAND *and* FRANCIS R. JAPP. *693 pages, octavo. Illustrated. Philadelphia:* LEA BROS. & CO. *Chicago:* JANSEN, MCCLURG & CO.

This work deserves more than a passing notice, although the names of the authors make this statement almost unnecessary. Men who have accomplished much as teachers, and whose scientific researches are of the highest order, are certainly in a position to write a book worthy of the careful study of any student, and a glance through the pages of the one before us is sufficient to show that we have here not a compilation, but a production of originality and of merit.

The book is intended for the use of students in our highest colleges and universities, and is a good introduction to the work of the specialist.

The peculiar formulæ which Frankland proposed some years ago, in his lecture notes, are here employed, and it will also be noticed that the subject of potable waters is treated somewhat more fully than one would expect in a general treatise.

